# Vision-Based Learning from Demonstration System for Robot Arms

**DOI:** 10.3390/s22072678

**Published:** 2022-03-31

**Authors:** Pin-Jui Hwang, Chen-Chien Hsu, Po-Yung Chou, Wei-Yen Wang, Cheng-Hung Lin

**Affiliations:** Department of Electrical Engineering, National Taiwan Normal University, Taipei 106, Taiwan; barry7577@gmail.com (P.-J.H.); 60875005h@ntnu.edu.tw (P.-Y.C.); wywang@ntnu.edu.tw (W.-Y.W.); brucelin@ntnu.edu.tw (C.-H.L.)

**Keywords:** learning from demonstration, action recognition, robotics, robotic arms, object detection, trajectory planning

## Abstract

Robotic arms have been widely used in various industries and have the advantages of cost savings, high productivity, and efficiency. Although robotic arms are good at increasing efficiency in repetitive tasks, they still need to be re-programmed and optimized when new tasks are to be deployed, resulting in detrimental downtime and high cost. It is therefore the objective of this paper to present a learning from demonstration (LfD) robotic system to provide a more intuitive way for robots to efficiently perform tasks through learning from human demonstration on the basis of two major components: understanding through human demonstration and reproduction by robot arm. To understand human demonstration, we propose a vision-based spatial-temporal action detection method to detect human actions that focuses on meticulous hand movement in real time to establish an action base. An object trajectory inductive method is then proposed to obtain a key path for objects manipulated by the human through multiple demonstrations. In robot reproduction, we integrate the sequence of actions in the action base and the key path derived by the object trajectory inductive method for motion planning to reproduce the task demonstrated by the human user. Because of the capability of learning from demonstration, the robot can reproduce the tasks that the human demonstrated with the help of vision sensors in unseen contexts.

## 1. Introduction

Nowadays, robotic arms have become more and more important in factory assembly lines [1,2] due to their ability to perform repetitive tasks with an accuracy of a few thousandths of an inch while being operable 24 h a day. Given the advantages, the costs of deploying robots to a product line, however, are much higher than the robots themselves. For example, the costs of system integration and deployment are generally 2–4 and 4–6 times as high as the robots’ cost for a new task. This has created a significant difficulty for companies targeting automation, particularly for small and medium-sized enterprises (SMEs). Thus, how to shorten robot programming time is one of the significant challenges in lowering the deployment costs for robots and establishing an automation system.

In low payload scenarios, programming by demonstration (PbD) [3,4,5] methods were introduced for industrial robotic systems by providing a hand-guiding mode that allows an end user to program the robot by physically dragging the robotic arm along desired locations in the workspace. No explicit programming skills or robotic expertise are required for the end user to teach desired tasks to the robot. This type of programming system not only helps companies lower the costs of deploying robots but also prevents downtime on production lines. By doing so, however, the robotic arms can only imitate the job based on record-and-replay macros through various types of sensors for encoding the motion of human demonstrators, such as kinesthetic teaching (or called hand-guiding) [6], data gloves [7], motion sensors [8]. As a result, these kinds of programming by demonstration can only mimic the tasks and they fail to understand the intentions behind the recordings and therefore will not be able to reproduce similar tasks in unseen contexts [9].

For a robot to learn a task effectively subject to environment changes, learning from demonstration (LfD) robotic systems were proposed [10,11,12,13,14,15,16] from the viewpoints of understanding and reproduction. For understanding the behavior demonstrated by a human, vision-based deep learning approaches using 3D convolution have been widely employed in action recognition architectures [17]. Among them, a deep learning-based multiple-person action recognition system [18] for use in various real-time smart surveillance applications was proposed using inflated 3D ConvNet (I3D) [19]. A two-stream architecture [20] taking both RGB and optical flow images as input data was revealed to improve the accuracy of action recognition by adopting the concept of Inception. Moreover, an abnormal activity recognition system from surveillance videos was revealed using CNN and Long Short Term Memory network (LSTM) models for spatial and temporal features extraction, respectively [21]. As far as actions in a LfD robotic system are concerned, we need to emphasize real-time subtle hand action recognition. Unfortunately, most existing action detection methods focus more on coarse human action detection, rather than fine-grained action detection of the hand movement. Furthermore, because 3D convolution relies on a huge amount of network parameters, existing network architectures inevitably lead to low efficiency in reaching a real-time action recognition. Moreover, it is important to understand the intentions or goals behind the demonstration by humans to reproduce a task when the environment changes. Early approaches tried to generalize the reproduction process by asking the teacher/demonstrator for further information [22,23] or using Hidden Markov Model [24,25,26]. Due to performance consideration, most of the research via kinesthetic teaching needed huge amount of data to learn the task for robot reproduction. As a result, even though the robot had learned the task, the robot still struggled to reproduce the behavior demonstrated by the human user in an unseen context.

As an attempt to address the problems of existing LfD robotic systems, we propose a novel vision-based LfD system for robotic arms incorporating two sub-systems: human understanding and robot reproduction. Both the understanding system and reproduction system can operate independently through an action base that serves as an interface between these two systems. In the human understanding system, a vision-based spatial-temporal action detection system was proposed by combining a single image and continuous video stream to improve the accuracy of action recognition for small and rapid hand movement of the demonstrator in real time. An object trajectory inductive method is then proposed to obtain a key path for objects manipulated by the human through multiple demonstrations. In robot reproduction, we integrate the actions in the action base and the key path derived by the object trajectory inductive method for motion planning to reproduce the task demonstrated by the human user. To this end, the contributions of the proposed work are summarized as follows:

- A spatial-temporal action detection system is proposed to deal with meticulous hand movement;
- Computational efficiency of the LfD system incorporating a 3D-2D network architecture is significantly improved for real-time action detection;
- A trajectory inductive method is proposed for analyzing the intention of tasks demonstrated by a human user without pre-programming of the robot motion;
- The LfD system can reproduce the task demonstrated by a human user in unseen contexts by integrating the sequence of actions in the action base and the key path derived by the object trajectory inductive method.

This paper is organized as follows. Section 2 shows the overall architecture of the learning from demonstration robotic system. Section 3 describes the understanding sub-system, including spatial-temporal action detection and action base. Section 4 presents an inductive trajectory method to analyze the intention of tasks demonstrated by a human user. Section 5 describes the robotic arm reproduction through motion planning based on the inductive trajectory method. Section 6 shows the experimental results. Finally, we conclude this paper in Section 7.

## 2. Architecture of the Learning from Demonstration Robotic System for Static Robot Arms

**Figure 1:**
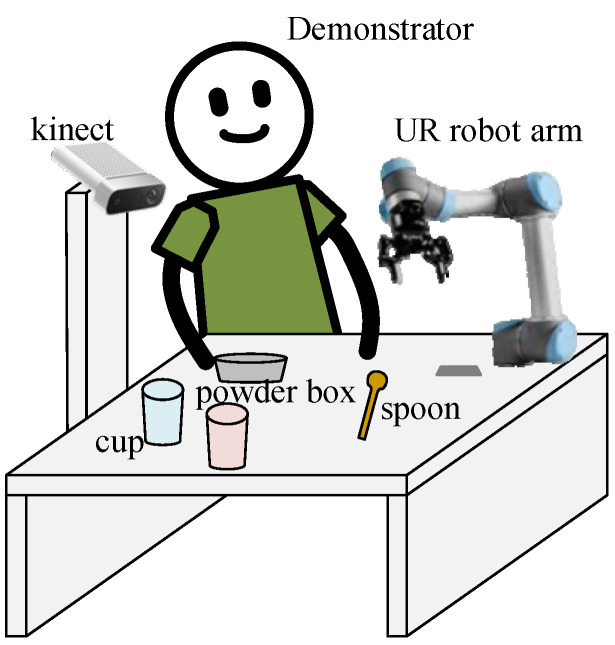
Experimental environment to exemplify the vision-based LfD robotic system.

To showcase the proposed vision-based LfD robotic system, an experimental environment is exemplified as shown in Figure 1, where the major components include a Kinect RGB-D camera, 6-DOF UR3 robotic arm, and PC-based controller. The camera is to capture video streams in the workspace, based on which the proposed spatial-temporal action detection can be used to understand the behavior demonstrated by a human user. For illustration purposes, we consider a scenario in a kitchen involving coffee or tea making activities, where actions demonstrated by a human user include holding (an object), pouring, stirring, scooping, adding in, etc.

**Figure 2:**
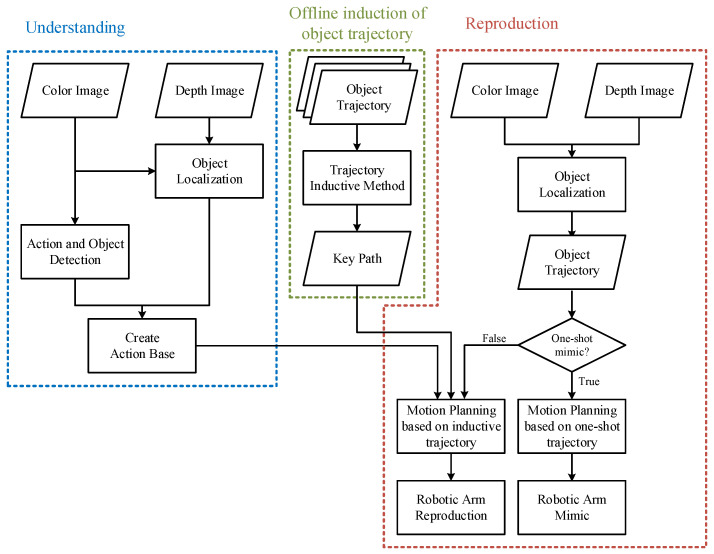
Flow chart of the proposed LfD robotic system.

Figure 2 shows the flow chart of the proposed LfD robotic system, which consists of 3 major blocks: understanding, reproduction, and offline induction of object trajectory. The understanding sub-system firstly captures color and depth images to locate the object being manipulated by the human. In the meantime, color images are used by the proposed spatial-temporal action detection and object detection methods to obtain the action classes and object classes, respectively. Together with the results obtained from object localization, we can create an action base to describe the behavior of the human demonstrator, including the action demonstrated, object being interacted with, and destination of the object.

Next, we can look at the reproduction system marked in red dashed lines, in which the object trajectory is obtained through object localization similar to that of the understanding system. To plan a motion for the robotic arm according to what the human user demonstrated, each action in the action base needs to associate with a key path to derive a trajectory for reproduction by the robot. The key path of an object being manipulated can be obtained by the proposed object trajectory inductive method marked in green dashed lines by considering a few shots of the object trajectory through multiple offline demonstrating/teaching by the human demonstrator. With the information of the action base and key path associated with each action, we can plan a motion for the robotic arm to reproduce the human task. In general, multiple demonstrations are required to obtain trajectories of the object being manipulated, based on which the key path can be obtained by the object trajectory inductive method. However, if the user only demonstrates the task once, the reproduction will be considered one-shot mimicking, where motion planning for the robot is performed according to the one-shot trajectory to mimic the task demonstrated by the user.

## 3. Understanding from Human Demonstration

### 3.1. Spatial-Temporal Action Detection

**Figure 3:**
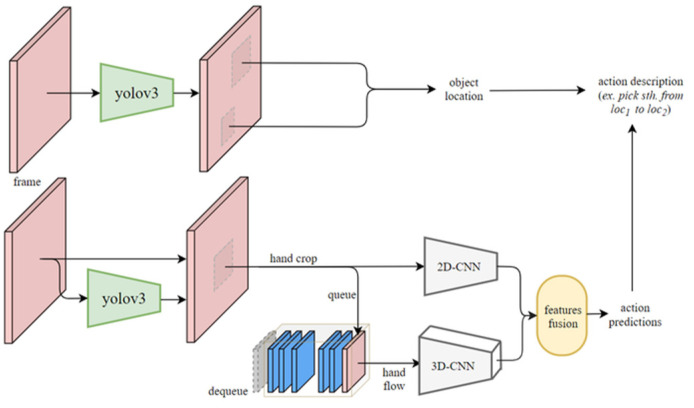
3D-2D network architecture associating action detection with object detection to create an action description in the action base.

In vision recognition, we propose a novel network architecture for recognizing small and fast hand action, such as stirring liquid in a cup or scooping powder in a jar. These actions are meticulous in finger or hand movement, which are difficult to recognize by existing action detection methods. For example, scooping powder is an action that only focuses on flipping the spoon. To recognize the subtle movement, a large network and sufficient amount of data are generally required. Unfortunately, it is generally difficult to recognize the actions in real time by a large network. To address the problem of computational efficiency, we propose a network architecture that combines two kinds of features, i.e., single image feature for hand gesture and multi-image feature for temporal action, as shown in Figure 3. We first capture color images from an RGB camera as input into a YOLO-v3 network to recognize hand and objects. The Region of Interest (ROI) results of hand, e.g., 16 frames, will be continuously inserted into a queue for processing by a 3D-CNN network that we propose. Based on the accumulated frames of the ROI, the 3D-CNN network can recognize the action class of the hand movement. At the same time, a 2D-CNN also predicts an action class of the hand movement based on the current frame of ROI. Merging the prediction results from the 3D-CNN and 2D-CNN via feature fusion, we thus obtain the final action predictions of the hand movement. The advantage of realizing the spatial-temporal action detection through the above method is that the 2D network provides static features that the 3D network fails to learn. Because our task focuses on action detection of fast hand movement, the proposed 3D-2D CNN architecture based on 3D-2D feature fusion can provide a satisfying computational efficiency with sufficient accuracy for action detection. Combined with the object detection results including object class and location from YOLO v3, we are able to associate the action class with the object being manipulated to create an action description in the action base.

**Figure 4:**
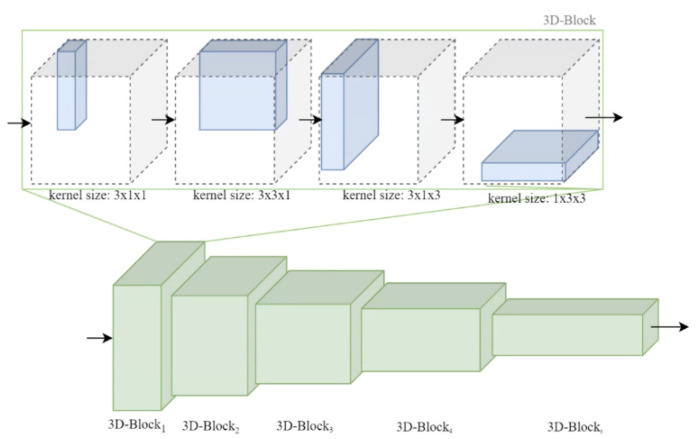
3D CNN constructed by five 3D-Blocks each consisted of 4 different 2D kernels.

It is understood that existing 3D-CNN networks, such as I3D or Resnet3D, for action recognition generally have a large number of parameters, which poses a heavy computation burden. As an attempt to solve this problem to increase the computational efficiency, we thus propose a 3D CNN constructed by five 3D-Blocks, as shown in Figure 4, where each 3D-Block is consisted of four different 2D kernels focusing on any two dimensions of time, height, width, to prevent the time-consuming computation of conventional 3D kernels. This change to the conventional 3D-CNN architecture can significantly reduce the parameters of the network, which will be illustrated later in the experimental results section.

### 3.2. Development of Action Base

As shown in Figure 2, the action base provides details of a task that a human user demonstrates by grouping three data items into a row for action description, including an action class, an object being interacted with, and a destination of the object. A full task can be divided into a few groups of action description through action detection, object detection, and object localization for adding into the action base according to the sequence in time. Take a painting task as an example. The user might firstly pick up a brush, dip the brush in a bucket, and then paint at a location. The complete action base for this task might look like Table 1, where the first action is “Pick” and the interacted object is “Brush,” indicating that the user picks up a brush. Notice that there is no destination in the first action because picking an object does not require a destination. The second action “Dip” describes how the user holds and dips the brush in a bucket. Unlike the first action, there is a destination “Bucket” for the second action to describe the user’s intention. Note that the destination can be an object or a location. In the third action description, the destination of the action “Paint” is a location coordinate, but it can also be changed into an object depending on the demonstration scenario. The action base established via action and object detection provides the details of a particular task, based on which the robotic arm can reproduce the behavior demonstrated by the user through motion planning.

## 4. Offline Induction of Object Trajectory

To remedy the problem of motion planning for an action through pre-defined trajectories [11,12], this method tries to find out the behavior of a task through a few shots of demonstration so that the robotic arm can reproduce the task. In other words, the objective of this method is to understand the intention of a task for motion planning without pre-defining motor angles for a corresponding action. 

To analyze the intention behind an action, we need to record position and its trajectories for each object. Based on the distance between objects and their position, we are able to induce the intention of an action. For example, if the action is “carrying a box with a cart”, we need both trajectories of the box and the cart to understand what the human user is carrying; otherwise, the action will more likely be understood as “moving the cart” because the distance between the box and the cart provides important information that cannot be ignored. As a result, three types of object trajectories are required to understand the intention of a task:

- Type-1: absolute trajectory of the object itself;
- Type-2: relative trajectory from the starting point of the object itself;
- Type-3: relative trajectory from other objects to the object itself.

Notice that each trajectory among the three types above has only one interacted object. If the interacted object is changed into another object in a task, the resulting trajectory needs to be saved into another segment. A task might include several segments in sequence, depending on how many times the demonstrator switches interacted objects.

In summary, a task that the robot requires to learn includes a few shots of demonstration referred to as ‘generation’. Each generation of the task has a number of segments. Each segment in a generation has only one interacted object and three types of object trajectory. The data structure of the trajectory inductive method is shown below:

- Task: List of generation
- Generation: List of segments
- Segment: Object paths
- Absolute trajectory $\left(P^{self}-P^{w}\right)$
- Relative trajectory $\left(P^{self}-P^{{self}_{0}}\right)$
- Relative trajectory $\left(P^{self}-P^{other}\right)$

Where $P^{self}$ represents the coordinate of the object itself when the object is moving, $P^{w}$ is the origin of the world coordinate system, $P^{self_{0}}$ is the coordinate of the starting point of the moving object itself, and $P^{other}$ represents the coordinate of other objects that do not move in the segment. Thus, $P^{self}-P^{w}$ means the path is relative to the world coordinate, indicating that the path is an absolute trajectory. On the other hand, $P^{self}-P^{{self}_{0}}$ and $P^{self}-P^{self_{0}}$ represent a relative trajectory with reference to $P^{{self}_{0}}$ and $P^{other}$, respectively.

To illustrate how the proposed trajectory inductive method works, we use a simulation environment to illustrate three different tasks where two objects, a blue rectangle and a green circle at arbitrary positions in the workspace, are manipulated by the user. These objects are manipulated to generate trajectories of types 1, 2, and 3 under the condition that only one object can be moved at a time. 

**Figure 5:**
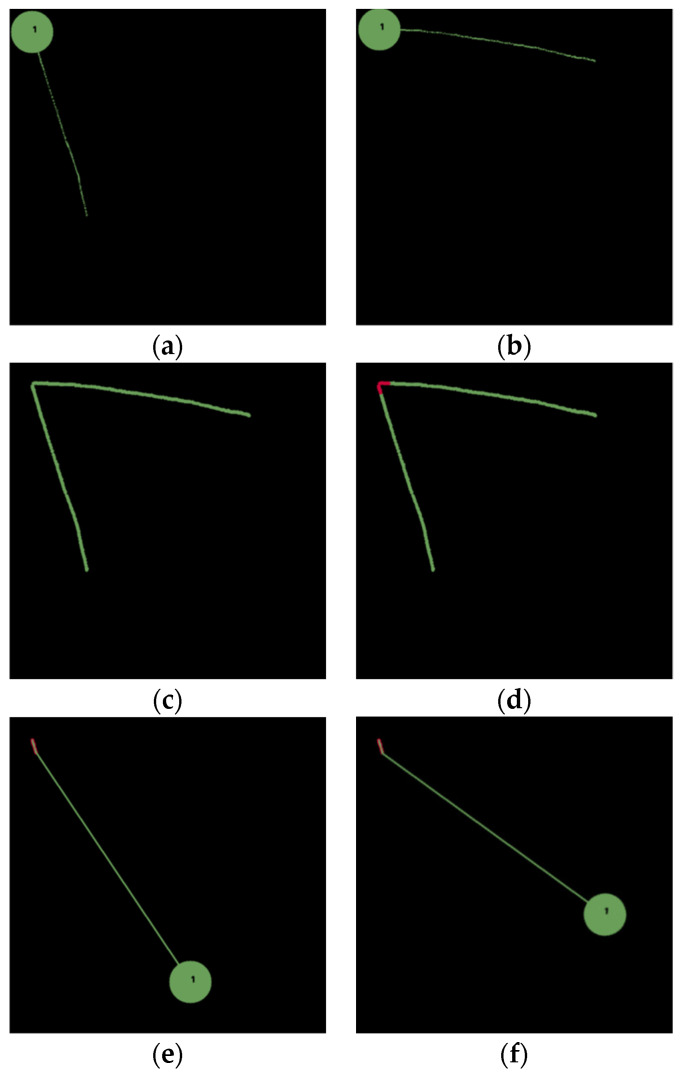
‘Pick & place’ a green circle at the top left corner. (**a**) The first demonstration path. (**b**) The second demonstration path. (**c**) Absolute trajectory (type-1) through two demonstrations. (**d**) Key path of the absolute trajectory (type-1). (**e**,**f**) Planned path based on the key path.

Figure 5 shows the first task, where the goal of the task is to pick & place a green circle to the top-left corner. Two demonstrations are conducted, as shown in Figure 5a,b, resulting in two absolute paths, as shown in Figure 5c. The inductive method first calculates distances between each point in a particular generation and all other points in other generations. If there exists a distance less than a threshold, then its corresponding point is added into the key path, resulting in the overlapped portions marked in red, as shown in Figure 5d. With the key path, we understand that the intention of this task is to pick & place an object to the top-left corner. Therefore, we can plan a path for the robot to pick & place a green circle at an arbitrary position, as shown in Figure 5e,f, to the top-left corner. This task exemplifies the use of the absolute trajectory to find a key path, based on which we understand the intention of the task to place an object at a particular position indicated by the key path.

**Figure 6:**
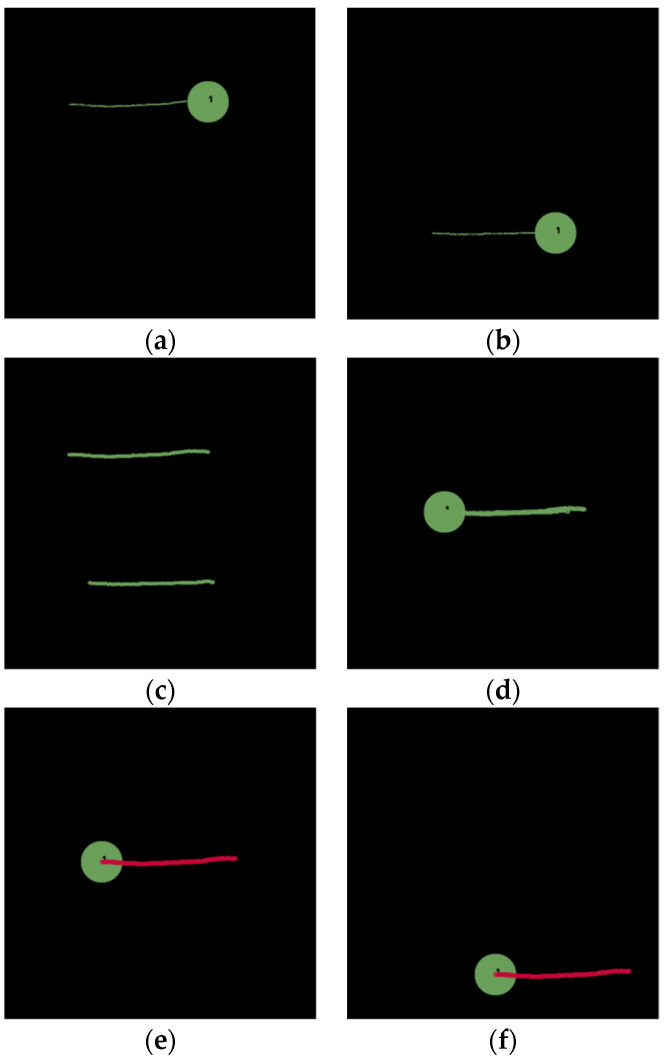
‘Push’ a green circle to the right side. (**a**) The first demonstration path. (**b**) The second demonstration path. (**c**) Absolute trajectory (type-1) through two demonstrations. (**d**) Relative trajectory (type-2) through two demonstrations. (**e**) Key path of the relative trajectory (type-2). (**f**) Planned path based on the key path.

In the second scenario, the goal of the task is to push an object to the right side, similar to swiping with a broomstick in a real-world scenario. Two demonstrations are conducted, as shown in Figure 6a,b. Based on the inductive method, the absolute path is shown in Figure 6c and a relative path relative to a starting position of the object is shown in Figure 6d. Finally, the overlapping portions marked in red in Figure 6e can be obtained as the key path, based on which a motion can be planned for the robot to push a green circle at an arbitrary location to the right, as shown in Figure 6f.

**Figure 7:**
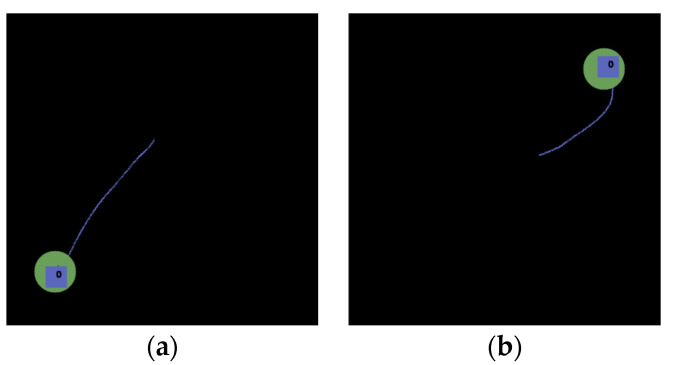

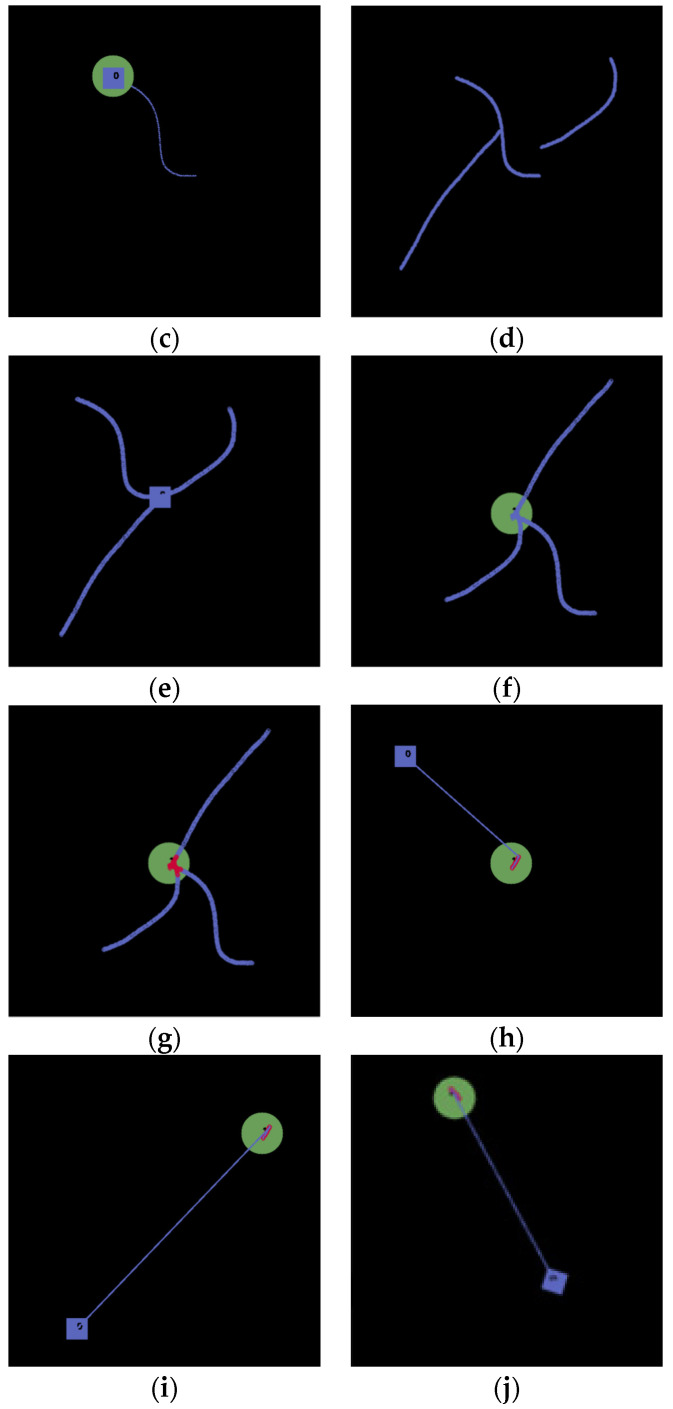
‘Pick and place’ a blue rectangle on a green circle. (**a**) The first demonstration path. (**b**) The second demonstration path. (**c**) The third demonstration path. (**d**) Absolute trajectory (type-1) through three demonstrations. (**e**) Relative trajectory (type-2) through three demonstrations. (**f**) Relative trajectory (type-3) through three demonstrations. (**g**) Key path of the relative trajectory (type-3). (**h**–**j**) Planned path based on the key path.

The last example is to pick and place a blue rectangle onto a green circle (i.e., another object). Three demonstrations are conducted, as shown in Figure 7a–c. Observing both the absolute path in Figure 7d and the relative path to a starting position of the object itself (i.e., blue rectangle) in Figure 7e, we found that there is no overlapping according to the three demonstration paths. However, a critical path relative to the green circle shown is shown in Figure 7f, resulting in a key path indicated in red, as shown in Figure 7g. As a result, a path can be planned to pick and place a blue rectangle at an arbitrary location onto a green circle at an arbitrary location, as shown in Figure 7h–j.

Notice that the method not only deals with the exemplary tasks with only one action introduced earlier, but also tasks involving a series of actions through the use of multiple types of trajectories to derive a key path. For example, the user might demonstrate a brushing task at a particular position that includes dipping a brush in a paint bucket at an arbitrary position, stirring in the paint bucket with the brush, and finally brushing at the position. Regarded as a single task, the method is still effective to determine a key path, although several actions are involved in this task. As can be seen from the above discussions, this method compares each demonstration path for generalization to induce a key path, based on which the robot arm can reproduce the task via motion planning. The derivation of the three types of trajectories mentioned earlier through multiple demonstrations can help obtain the correct overlapped path to clarify the intention of the task. As a result, the robot can avoid unnecessary movement because of the key path.

## 5. Robotic Arm Reproduction

### 5.1. Motion Planning Based on Inductive Trajectory

As soon as an action base is created, the next step is to plan a motion for the robot arm for each action in the action base. To plan the motion for each action, the system firstly recognizes the object and locates the object through visual recognition. Second, a path connecting the starting position and each position on the key path can be derived as the overall reproduction path of the task. Next, we transform the reproduction path from camera space into robot arm tool space through an extrinsic matrix in order to plan a trajectory of the end-effector of the robot to reproduce the task. As soon as the motion trajectory in the robot arm tool space is derived, we calculate the joints angle for each axis of the UR3 6DOF robotic arm through inverse kinematics. 

A PC-based controller is responsible for handling all of the processing, including object detection, action detection, creating action base, object trajectory induction, and control commands of the UR3 robotic arm.

### 5.2. One Shot Mimic

Thanks to the object trajectory inductive method, we can find out the key path from multiple demonstrations of the user. However, if the task was demonstrated only once from the demonstrator or the task cannot be successfully detected by the action recognition module, the key path under this circumstance would be the full trajectory of the demonstration for the robot arm to reproduce the task. With the key path, the robot can still go through the motion planning process to mimic the task that the human demonstrated. However, because the key path is the full trajectory of the demonstration, the robotic arm has no choice but to follow the full trajectory of the demonstration. This might result in unnecessary movements of the robotic arm, which does not reflect the true behavior of the task. Furthermore, when the robot is mimicking, the starting position of the object in the environment has to be the same position as that which the human demonstrated. If the starting position of the object is different from the position that the human demonstrated, we need to firstly plan a path to the starting position that the human demonstrated earlier, then follow the same trajectory that the human demonstrated.

## 6. Experimental Results

**Figure 8:**
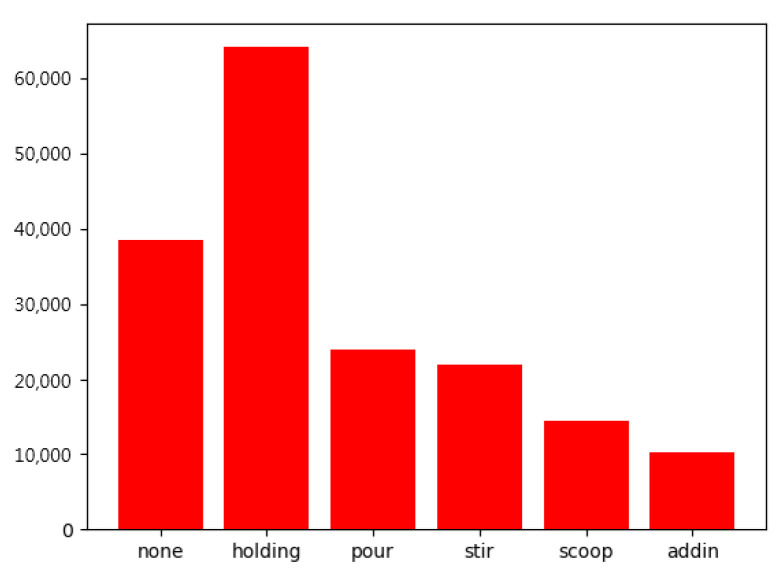
Uneven data in each action class.

To validate the proposed LfD robotic system, we use a scenario in a kitchen involving coffee or tea making activities, where a LfD data set created by ourselves consisting of six action classes, include holding (an object), pouring, stirring, scooping, adding in, and none, will be used for training the deep-learning network. The computational platform that we used to verify the proposed method is a desktop computer with an AMD Ryzen 7 3700X 8-Core Processor @ 3.6GHz, 32G RAM and a NVIDIA GeForce RTX 2080ti graphic card under Windows 10. The experiments are conducted using Pytorch toolbox as the main implementation substrate and NVIDIA CUDA 10.2 library for parallel computation. There are 480 videos for each action class in total of the LfD data set. Due to practical implementation during demonstrations, the LfD dataset that we created has uneven data in each class, as shown in Figure 8. Therefore, we use focal loss [27] to further improve the accuracy.

Table 2 shows a performance comparison of backbone architectures for action recognition of the LfD dataset. We can see that the proposed 3D-2D CNN network has a competitive mAP of 93.9 with a relatively small parameter size of 65.04 M and 65.48 G floating point operations (FLOPs) for input image size of 112 × 112. Thanks to the small architecture of the proposed 3D-2D CNN network, the processing time for action recognition is around 6.8ms, reaching the best computational efficiency of 146.96 frames per second in comparison with the state-of-the-art models. Because of the high computational efficiency of the proposed model, the behavior demonstrated by the user can be detected in real time, which is indispensable for LfD robotic systems.

**Figure 9:**
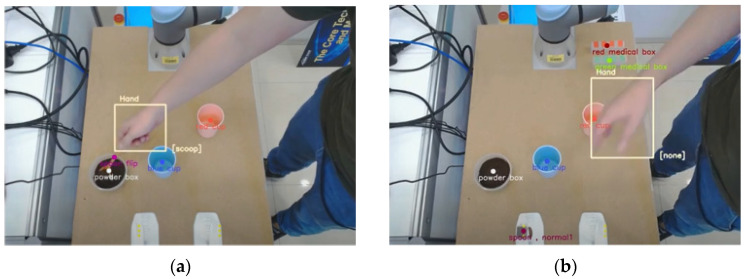
Experimental results of spatial-temporal action detection. The hand position is indicated by a yellow box and the action class is shown at the bottom left of the box. (**a**) example of detecting action “scoop”. (**b**) example of detecting nothing ‘none’.

As a practical experiment, Figure 9 illustrates the understanding system via the proposed action and object detection using an Azure Kinect camera, where bounding boxes indicating hand and objects are displayed. Furthermore, the action class of the hand movement and the object being manipulated are also indicated in Figure 9, where the hand position is indicated by a yellow box and action class occurring in that moment is displayed in the lower right corner of the box. In addition, the positions of the target objects, such as cups, are also represented by color dots. Note that the Azure Kinect camera has a frame rate of only 30 fps to capture images. As a result, the processing time of the overall system in a practical real-world scenario is about 39.8ms, achieving an execution speed of 25 fps in real time.

**Figure 10:**
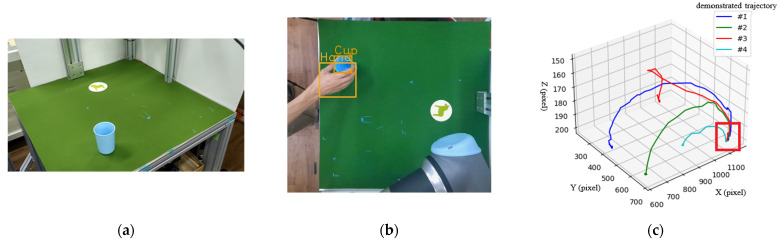
Scenario 1: “pick & place a cup to a fixed position”. (**a**) Experimental environment; (**b**) object detection results; (**c**) absolute trajectory (type-1) of the cup through four demonstrations to induce a key path indicated by a red box.

To show how the object trajectory inductive method practically works, we set up an experimental environment to observe the object’s trajectories. In the first scenario, the task is to pick & place a blue cup to a particular position, i.e., the coaster on the table, as shown in Figure 10a,b, where the cup and hand are detected by the object detection method. Notice that the coaster is not detected as an object. The use of the coaster is only for humans to correctly pick & place the cup at the same position in each demonstration. Figure 10c shows the absolute trajectory (type-1) of the cup through four demonstrations, based on which we can induce a key path in the right-bottom corner of the figure marked by a red rectangle. The other types (type-2 and type-3) of trajectories mentioned in Section 4 do not result in any key path. Thus, they will not be used in this scenario. With the help of the key path, we can plan a motion for the robot arm to reproduce the task.

**Figure 11:**
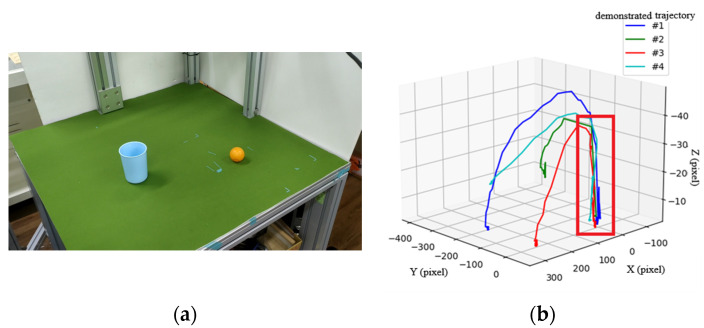
Scenario 2: “place a ball into a cup”. (**a**) Experimental environment; (**b**) relative trajectory (type-3) from the cup to the ball through 4 demonstrations to induce a key path indicated by a red box.

The second scenario is shown in Figure 11, where a user places a ball into a cup, all with random positions. Through four demonstrations, type-3 trajectories from the cup to the ball are shown in Figure 11b, in which a key path indicated by a red box at the right corner of the figure is induced by the proposed method.

**Figure 12:**
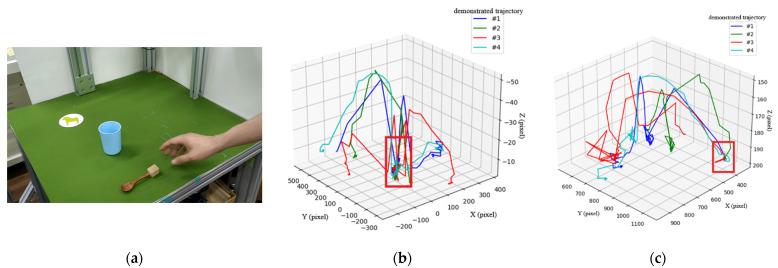
Scenario 3: “pick up a spoon, stir in a cup, and place the spoon at a fixed position”. (**a**) Experimental environment; (**b**) relative trajectory (type-3) from the cup to the spoon through four demonstrations to induce a key path indicated by a red box; (**c**) absolute trajectory (type-1) of the spoon through four demonstrations to induce a key path indicated by a red box.

The third scenario shown in Figure 12 is a more complicated task, including picking up a spoon, stirring in a cup, and placing the spoon at a particular position (the coaster). Figure 12b,c show the type-3 and type-1 trajectories, respectively, after four demonstrations, where key paths are indicated by a red rectangle. Note that the type-2 trajectory results in no key path and is therefore ignored and not included here. Moreover, the type-3 trajectories in Figure 12b are used to derive the key path for the action of stirring in the cup, while type-1 trajectories in Figure 12c are used to derive the key path of placing the spoon to a fixed position. According to the key path that we have obtained, motion planning of the robot arm can be performed to first pick up a spoon at an arbitrary position, then stir in a cup at an arbitrary position, and finally place the spoon at the fixed position. 

To summarize, the method can identify the intention behind an action through multiple demonstrations, helping the robot to reproduce the task involving objects at arbitrary positions using three types of trajectories. Interested readers can refer to the following link: http://isdlab.ie.ntnu.edu.tw/isdwebsite/research_en.html (accessed on 1 March 2022) or https://youtu.be/M-WwKeNMqOE (accessed on 28 February 2022) to view the video clip showing human understanding and robot reproduction using the proposed approach in this paper.

## 7. Conclusions

In this research, we presented a novel LfD robotic system to address the problems of understanding through human demonstration and reproduction by robot arm. From the understanding point of view, we proposed a spatial-temporal action detection system that takes account of a single image and continuous video stream to recognize meticulous hand actions during human demonstration to create an action base. Thanks to the proposed 3D-2D CNN architecture consisting of different 2D kernels, the computational efficiency is significantly improved because of the lightweight architecture while maintaining a satisfactory accuracy. Thus, the behavior demonstrated by the user can be detected in real time, which is extremely important for LfD robotic systems. From the reproduction point of view, we proposed a trajectory inductive method to analyze the intention of tasks demonstrated by the human user to derive a key path through multiple demonstrations. Integrating the sequence of actions in the action base and the key path derived by the object trajectory inductive method, we can plan a motion for the robot to reproduce the task demonstrated by the human user in unseen contexts without pre-programming.

To further improve the performance of the LfD system, however, there are several issues that deserve further investigation in the future. Firstly, the current system does not consider object pose nor grasping pose because the object detection module does not provide the object’s orientation. Grasping of objects at this stage, however, relies on pre-defined specific orientation of the objects. To successfully grasp an object with any pose, a suitable grasping strategy needs to be developed with the help of an object pose estimation algorithm. Secondly, the object trajectory inductive method aims to obtain a key path for objects manipulated by the human, based on which a trajectory can be planned for the robotic arm. To allow a safe reproduction, the trajectory for the robot should avoid any collisions over time. Thus, a collision-free trajectory planning needs to be considered in the future. Considering that dynamical obstacles might occur while the robotic arm is moving, we also need to design a suitable obstacle avoidance algorithm to eliminate the risk of collision.

## Figures and Tables

**Table 1:** Action base illustrating a painting task.

Action	Interacted Object	Destination
Pick	Brush	-
Dip	Brush	Bucket
Paint	Brush	Location coordinate

**Table 2:** Performance comparison of backbone architectures for action recognition of the LfD dataset.

Backbones	Parameters (M)	Frames/S	mAP_50
I3D-50	47.3	58.97	94.6
Resnet3D-50	177.0	30.67	94.2
R(2 + 1)D-50L	176.36	38.31	94.5
C3D	297.66	74.62	92.5
R(2 + 1)D-5L	54.96	125.31	93.8
Proposed	65.04	146.96	93.9

## Data Availability

Not applicable.
